# Overview of Histone Deacetylase Inhibitors in Haematological Malignancies

**DOI:** 10.3390/ph3082674

**Published:** 2010-08-17

**Authors:** Mark J. Bishton, Ricky W. Johnstone, Michael Dickinson, Simon Harrison, H. Miles Prince

**Affiliations:** 1 Cancer Immunology Research, Peter MacCallum Cancer Centre, Melbourne, Australia; 2 Department of Haematology and Medical Oncology, Peter MacCallum Cancer Centre, Australia; 3 University of Melbourne, Parkville, Melbourne, Australia

**Keywords:** Histone deacetylase inhibitors, Clinical studies, Haematology

## Abstract

Histone deacetylase inhibitors (HDACi) can induce hyperacetylation of both histone and non-histone target resulting in epigenetic reprogramming and altered activity, stability and localisation of non-histone proteins to ultimately mediate diverse biological effects on cancer cells and their microenvironment. Clinical trials have demonstrated single agent HDACi to have activity in hematological malignancies, in particular T-cell lymphoma and Hodgkin lymphoma. Combination strategies with standard therapies based on pre-clinical data are being employed with significant success due to their excellent side effect profile. Correlative studies will provide valuable information on the sub-groups of patients more likely to respond or be resistant to HDACi therapy, while long-term monitoring for toxicities is also needed.

## 1. Introduction

Histones are a family of proteins that interact with DNA resulting in DNA being wound around a histone octameric core within a nucleosome. The organization of chromatin affects key molecular processes such as gene transcription, DNA repair, recombination and replication [1], and multiple post translational modifications occur at specific sites within the histone N-termini, regulating chromatin structure and DNA accessibility. The histone deacetylase (HDAC) enzymes are a multi-class, multi-member family that affect chromatin conformation via deacetylation of ε-N-acetyl lysine amino acids on histone proteins [2]. They are highly conserved and are classified based by their homology to yeast enzymes [3]. A total of 18 HDACs have been described, divided into four general classes. Class I includes the HDACs 1, 2, 3 and 8, located within the cell nucleus; class IIa (with one catalytic site) includes the HDACs 4, 5, 7 and 9; class IIb (with two catalytic sites) includes the HDACs 6 and 10; and the class IV HDAC, HDAC 11. Class IIa, IIb and IV HDACs shuttle between the cell cytoplasm and nucleus. Class III HDACs consist of the NAD+-dependent sirtuin family 1 to 7 [4]. HDACs work in opposition to histone acetylase transferase enzymes (HAT), which result in net gain in lysine acetylation. HDACs and HATs control the degree of histone acetylation and deacetylation which, in concert with other epigenetic changes such as methylation, phosphorylation and sumoylation, facilitates open or closed chromatin states. Increased acetylation of histones is associated with open DNA and increased transcription, and deacetylation with transcriptional repression [5]. Epigenetic changes play a fundamental role in cancer onset and progression, with hypermethylation of tumor suppressor gene promoter sites and resultant transcriptional silencing, occurring in conjunction with global DNA hypomethylation commonly seen in cancer cells [6]. These epigenetic changes are reversible and thus valid targets for anti-cancer therapy [7].

## 2. HDAC Inhibitors (HDACi)

The HDACi’s are a new class of structurally diverse, targeted anti-neoplastic agents. These drugs are all able to bind to the catalytic pockets of class I, II and IV HDACs [8], and can be subdivided into six groups based on their highly disparate chemical structures. It is unknown whether these structural differences result in different capacities to hyperacetylate lysine residues on histone and non-histone targets, and pharmacokinetic properties, or if targeting HDAC isotypes using class I selective drugs such as mocetinostat or entinostat results in responses equivalent to pan-HDACi, with a potential reduction in toxicity [9,10,11]. Moreover, acetylation of non-histone proteins such as P53 by these drugs results in alterations in their function, and downstream effects on tumor cells [8]. Nevertheless, despite these unknowns these drugs have rapidly entered the clinical arena and shown efficacy in multiple malignancies, in particular hematological cancers. Given the increasing and large number of HDACi in pre-clinical and clinical development, breadth of depth of pre-clinical and clinical studies in a huge number of diseases, for the purposes of succinctness this review will concentrate upon the clinical studies of the more developed HDACi in hematological malignancies alone.

## 3. Vorinostat

Vorinostat (suberoylanilide hydroxamic acid, SAHA), a hydroxamic acid derivative, is an analogue of trichostatin A and has pan-HDACi effects. The US Food and Drug Administration (FDA) approved vorinostat for the treatment of relapsed or refractory cutaneous T-cell lymphoma (CTCL) [12]. The therapeutic use of both intravenous and oral formulations of vorinostat came to light with the combined reporting of separate phase I studies in patients with advanced malignancies [13]. At a variety of dosing intensities, responses were observed in five patients with Hodgkin lymphoma (HL), diffuse large B-cell lymphoma (DLBCL), and CTCL. Discrepancies in both C-max and area under the curve (AUC) were evident between the methods of administration, although failed to account for the greater hematologic toxicity seen with oral vorinostat or the increased fatigue, infection and diarrhea seen in hematological patients generally. Increased histone hyperacetylation, a biomarker for the effects of HDAC inhibition, was seen in peripheral blood mononuclear cells (PBMCs) by two hours post treatment, with higher doses resulting in a longer duration of hyperacetylation. Histone hyperacetylation was also evident in tumor biopsies, although no correlation between hyperacetylation and response was seen, suggesting potentially numerous other targets affecting response which may impact on anti-tumor efficacy. A subsequent phase II study focusing on DLBCL showed only few responders, with one complete remission (CR), and one sustained stable disease (SD) [14]. A phase I study performed in patients with low grade lymphoma, reported two CRs/unconfirmed (CRu) and one partial response (PR) among patients with follicular lymphoma, and one CRu in a patient with mantle cell lymphoma, with one response maintained for 18 months [15]. Responses in low grade lymphoma were confirmed in a phase II study involving 37 patients, most with follicular lymphoma, with six patients achieving a CR and four PR, for an overall response rate (ORR) of 29%. There was a median progression free survival (PFS) of seven months, with five patients remaining on study for more than 18 months [16].

Following the surprising response of a patient with CTCL to vorinostat, 33 patients with relapsed/refractory CTCL were accrued to a single centre phase II dose finding study [17]. The ORR was 24%, with a reduction in pruritus seen in 58% of patients, allowing evaluation in a phase IIb multi-centre trial. This non blinded single arm pivotal trial in CTCL assessed responses by changes in overall skin disease score using a modified severity-weighted assessment tool (mSWAT; *ref.* 20). The objective response rate was 30% in 74 patients, with the most common toxicities gastrointestinal and hematological abnormalities, generally mild to moderate in severity. Skin biopsies taken prior to treatment to identify predictors of vorinostat response [18], suggested nuclear accumulation of signal transduction and activators of transcription (STAT) 1 (STAT1) and phosphorylated STAT3 in malignant T cells on immuno-histochemistry correlated with a poor response to treatment. This may potentially allow a routine immunostain to predict clinical responses to vorinostat. Similarly a recent paper identified levels of HR23B, which is involved in the transport of ubiquitinated proteins to the proteasome, as a marker for response to vorinostat. Preclinical work was validated using immuno-histochemistry on skin biopsies from patients treated with vorinostat on study, with high HR23B levels predicting response [19].

A dose escalating study of vorinostat in 41 patients with acute myeloid leukemia (AML) reported four patients (17%) to respond, including two who achieved a CR [20]. Grade 3/4 adverse events were predominantly fatigue, diarrhea, and thrombocytopenia. Rapid histone hyperacetylation was observed in PBMC’s and bone marrow in all patients, with levels returning to baseline during breaks in treatment. Gene expression analysis of PBMC’s validated previous pre-clinical work suggesting vorinostat efficacy may in part depend on the generation of reactive oxygen species (ROS) [21]. Genes associated with clinical resistance to vorinostat in this small study included overexpression of p21- and p53-responsive genes [22]. A phase II study failed to confirm the efficacy of single agent vorinostat in refractory AML, with more than half of 37 patients receiving fewer than two treatment cycles, and only one patient responding to treatment albeit for greater than one year [23]. Combination studies have been initiated, with the addition of both induction and maintenance vorinostat with idarubicin (Ida) and cytarabine (ara-C) as front line therapy in elderly patients with AML or higher risk myelodysplastic syndrome (MDS). From 45 evaluable patients there was no increase in toxicity compared to the historical comparator of idarubicin and cytarabine alone, and despite short follow up, the disease free survival (DFS) was a promising 9.5 months. Correlative studies showed LC-3, a marker of autophagy, ROS activation and NF-Kappaβ signalling to be upregulated [24].

Despite the lack of clinical studies of single agent vorinostat in multiple myeloma (MM), preclinical studies in combination with the proteasome inhibitor bortezomib has demonstrated significant synergy in the induction of apoptosis by plasma cells [25]. Mechanisms of bortezomib resistance include misfolded proteins accumulating following proteasome inhibition utilising alpha-tubulin, to form an aggresome in malignant plasma cells. HDACi induced tubulin hyperacetylation prevents aggresome formation, resulting in an increase in apoptosis [26]. More recently, bortezomib has been shown to down-regulate the expression of class I HDACs in MM cells at the transcriptional level, also resulting in an increase in histone hyperacetylation [27,28]. Phase I studies suggest the combination to be well tolerated, with near maximum single agent doses of both drugs deliverable. From twenty-three heavily pre-treated patients the overall response rate was 42%, including three partial responses among nine patients previously refractory to bortezomib. Despite this, protein levels of NF-kappaB, IkappaB, acetylated tubulin, and p21^CIP1^ on plasma cells at baseline and day 11 showed no correlation with clinical response [29].

## 4. Romidepsin

Romidepsin (depsipeptide) becomes an active compound following reduction of a disulphide bond following cell entry, becoming capable of preferentially interacting with the zinc in the active site of the HDAC class I enzymes. Romidepsin is still able to inhibit class II enzymes, but to a lesser degree [30]. The first published study in haematological malignancies undertaken with romidepsin was performed in patients with chronic lymphocytic leukemia (CLL) and AML patients, initiated with an goal to increase histone acetylation of by 100% *in vitro* [31]. Despite the lack of formal responses anti-leukemic effects were noted with reductions in peripheral leukemic cell number, with one patient experiencing tumour lysis. 

Following responses in four patients with T-cell lymphoma in a phase I trial [32], two large groups have investigated romidepsin in phase II trials. On the basis of data from these multi-centre, single-arm studies, of a total of 167 patients, romidepsin has recently received FDA approval for the treatment of refractory CTCL at a dose of 14 mg/m^2^ as a 4-hour infusion on days 1, 8, and 15 of a 28-day cycle with a starting dose of 14 mg/m2. Similar response rates were seen both studies (34% and 35% respectively), with a CR rate of 6% in both. The median response was 15.1 months and 11 months. Notably CRs were achieved even in patients with the leukemic form of CTCL, Sezary syndrome. Several patients also achieved very long durations of response, even after drug cessation, with one patient in a CR off therapy after 63 months [33,34]. Unlike vorinostat, correlative studies on samples from 61 patients from an NCI study did show an association of pharmacological alterations in genetic targets with response to treatment. Increases in the levels of histone acetylation and ABCB1 gene expression in PBMCs, as well as ABCB1 gene expression in tumor biopsy samples increased in all samples following romidepsin treatment. Moreover, the degree of histone acetylation in PBMCs at 24 hours did loosely correlate with the C-max and area under the curve, and was strongly associated with clinical response [35]. Microarray analysis revealed specific CTCL signature genes (which have been recognized to differentiate between Sezary syndrome and aleukemic forms of CTCL) were reversed following romidepsin treatment [36], suggesting either effective peripheral blood tumor elimination or differentiation. 

Similarly impressive single agent activity has been reported in patients with relapsed or refractory peripheral T-cell lymphoma (PTCL), with an ORR of 33% as a single agent in 46 patients. Responses included five CR’s and 10 PR’s with a median duration of response for all patients of 9 months. Responses appeared to be independent of prior therapy and subtype of PTCL [37]. 

Following concern over alterations in patient ECG traces, in particular QTc prolongation, recorded after romidepsin treatment, intensive electrocardiogram and ejection fraction monitoring were initiated in clinical trials involving this drug. A cardiac safety monitoring plan agreed with the FDA reported on 4909 ECGs from 110 patients in three clinical studies. Increases in the QTcF peaked 2 hours post infusion and returned to normal by 24 hours, with no patient increasing QTcF by more than 60 msec from baseline or having an absolute QTcF > 480 msec [38]. Therefore, careful patient selection, excluding those with a significant cardiac history, and the use of aggressive electrolyte replacement would appear to minimize any risk of QTc prolongation. Other common adverse events reported in the phase II studies were nausea, fatigue, vomiting, and cytopenias. 

Single-agent romidepsin has been trialled in MM, with no clinical responses, although some patients obtained SD with demonstrable decreases in monoclonal protein [39]. Following a similar rationale to the vorinostat/bortezomib studies, an ongoing Australian phase I/II trial is examining the combination of induction and maintenance romidepsin in conjunction with bortezomib and dexamethasone. Of 22 patients treated to date, there has been four CR, two very good partial responses (VGPR), six PR, five minimal responses (MR), and one SD. Patients refractory to prior bortezomib have also shown responses [40]. 

## 5. Panobinostat

Panobinostat (LBH589) is a structurally novel cinnamic hydroxamic acid analogue, with both intravenous and oral formulations. The original two-arm, dose-escalation phase IA/II used a seven consecutive day dosing schedule of intravenous panobinostat study in patients with advanced hematological malignancies. Following the detection of asymptomatic grade 3QTcF prolongation in four out of 15 patients resulted in premature discontinuation of the study. During later drug development it became clear that drug scheduling greatly influenced QTcF effects, and all subsequent studies have since used intermittent dosing schedules with minimal cardiac complications [41]. 

The first study of oral panobinostat on a Monday, Wednesday, and Friday (MWF) schedule on a dose escalating weekly and alternate weekly treatments enrolled 10 CTCL patients, with two achieving CR, four PR, two SD, and two patients PD [42]. The MTD was determined to be 20 mg for the MWF every week schedule due to dose-limiting diarrhea and thrombocytopenia-significantly lower than in other trials utilising this drug-in what appears a disease specific phenomenon. Histone acetylation occurred in normal PBMCs at doses of 15 mg and above for at least 72 hours in 50% of patients. Gene expression profiling performed on correlative skin biopsies from six patients at 0, 4, 8, and 24 hours post drug, showed the large majority of affected genes were down-regulated. A total of 23 genes were commonly up- or down-regulated in all patients, implicating cell cycle, cell proliferation, angiogenesis, and immune regulation as HDACi targets [43]. A phase II study of oral panobinostat delivered MWF in patients with refractory CTCL has enrolled 95 patients to date, with 15 demonstrating responses by SWAT and CT criteria [44]. 

Interim analyses of a large, ongoing, two-arm phase IA/II dose-escalation study has examined the role of panobinostat in several hematological diseases at multiple dose levels and weekly MWF or alternate weekly MWF dosing. This study has already enrolled 176 patients with doses ranging from 20 mg to 80 mg [45]. Panobinostat increased histone acetylation in PBMCs and bone marrow core biopsies relative to baseline at all doses over 20 mg, however the relationship between acetylation status and tumor response remains unclear. If the arms of the trial are analysed by disease, impressive responses have seen in both myeloid and lymphoid diseases. For example, in AML, once patients were treated at doses at and above 40 mg MWF weekly clear anti-leukemic activity was seen in seven of 36 evaluable patients with two CRs, one CR incomplete (CRi), and four patients with greater than 50% reduction in bone marrow and/or peripheral blood blasts seen. 

Results are available in twelve evaluable patients with myelofibrosis, eight of whom were transfusion dependent at study entry, and most had received prior treatments most commonly hydroxyurea. Nine patients had confirmed JAK2^V617F^ mutations. One previously untreated patient achieved a partial response, with an 85% reduction in spleen size, while three other patients have shown clinical improvement lasting ≥8 weeks including reductions in spleen size and transfusion independence [46]. These results are similar to those from another study using panobinostat for MF, with a total of 12 patients have been enrolled with eight evaluable patients. Of these, two patients have attained a PR with one transfusion independent, with a further four patients experiencing clinical improvement [47].

Panobinstat demonstrated striking effects against HL, by both positron emission tomography (PET) and computed tomography (CT) scanning, with one out of 28 patients attaining a metabolic CR, 16 PR, and eight SD, and one anatomical CR, nine PR, and 12 SD with two patients on therapy for more than 18 months. Constitutional symptoms were also relieved by the drugs, with results leading to the initiation of an international multicenter trial for relapsed refractory HL [45]. 

Preliminary results have been reported from a phase II study in which 38 patients with MM were treated with oral panobinostat 20 mg MWF with modest results [48]. As with other HDACi, studies have been initiated combining panobinostat with bortezomib, and a phase 1b study has already enrolled 29 patients in four cohorts with two dose levels for each drug. Fourteen responders with PR or better have already been seen in 28 evaluable patients, including four with immunofixation (IF) negative CR. A further four patients achieved minor responses, resulting in a 64% overall response rate. Again HDACi therapy was able to overcome prior bortezomib resistance in six of ten patients. Predominent adverse events were thrombocytopenia as well as other hematological abnormalities and fatigue [49].

There has been longstanding interest in the use of HDACi in chronic myeloid leukemia (CML), a disease driven by the deregulated oncogenic tyrosine kinase Bcr-Abl, primarily as Bcr-Abl is a client protein for the chaperone heat shock protein (hsp) 90. HDACi that inhibit HDAC6 are able to induce the acetylation of Hsp90, inhibiting association with client proteins, causing their subsequent degradation. Despite this and proof of concept in pre-clinical studies, early clinical results have not as yet borne out this promise. In phase I studies of panobinostat, only one patient achieving a hematological remission, with eradication of the T315I clone, but there were no other complete cytogenetic or molecular responders [50,51]. Poor outcome may relate to the low dose of 20mg MWF used in both studies, the TKI refractory nature of the disease. In this case, potentially the drug may be able to suppress the TKI resistant T315I clone and benefit patients in conjunction with standard TKI therapy.

## 6. Belinostat

Belinostat (PXD101) also belongs to the hydroxamic acid class of HDACi, and has been administered in a phase I study in patients with advanced B-cell malignancies as an infusion on days 1 to 5 of a 21-day cycle [52]. Sixteen patients were treated with disease stabilization occurring in five. Histone hyperacetylation of PBMC’s occurred rapidly, although was brief, with the duration related to dose. 

A phase II multi-centre trial treated 53 patients with relapsed PTCL and CTCL with the same dose schedule of belinostat described above. Five of 20 PTCL patients responded with two CRs and three PRs, with a median duration of response of 159 days, and a further SD in five patients. Regarding CTCL, four of 29 patients responded with two CRs and two PRs with a median duration of response of 273 days, and SD in 17 pts. Improvement in pruritus was seen in seven of 14 pts with significantbaseline pruritis. The drug was extremely well tolerated with few grade 3/4 toxicities [53]. Studies using belinostat as a single agent in both MM and lymphoma have been reported with both reporting patients having stable disease on treatment [54,55].

A study in AML added escalating doses of belinostat to azacytidine for advanced myeloid malignancies, predominantly relapsed or refractory AML. The combination was well tolerated, and of 21 evaluable patients there were two CRs, one PR and four with hematological improvements, most responders at the 1000 mg/m^2^ dose level. Another study in AML added belinostat to idarubucin in patients with AML not able to tolerate intensive therapy with no obvious elevation in toxicity [56].

## 7. Mocetinostat

Mocetinostat (MGCD0103) is a highly selective aminophenylbenzamide able to strongly inhibit HDAC classes I and IV, but with little class II effect. Due to its prolonged pharmacodynamics, the drug requires less frequent dosing [57]. Although development was temporarily suspended due to concerns over pericardial toxicity, occurring in around 4% of patients, mocetinostat has received orphan drug designation for HL and AML, with patients receiving the drug undergoing electrocardiogram and echocardiogram testing prior to initiation. Other common adverse events with MGCD0103 include fatigue and gastrointestinal symptoms, with little neutropenia and thrombocytopenia reported, albeit using protocol specific definitions of hematological toxicity [10]. The multi-centre phase 1 trial of oral MGCD0103 established 100 mg thrice weekly in 23 patients with leukemia and myelodysplastic syndromes as the maximum tolerated dose, with three patients responding [10]. A dose dependant acetylation of histone H3 was seen. Further studies in AML have used dose escalating thrice weekly mocetinostat in combination with the hypomethylating agent 5-azacytidine. In a phase I/II study, 37 evaluable patients, there were 11 objective responses including four CRs, five CRs with incomplete peripheral count recovery (CRi), and two PRs. Higher response rates occurred in patients receiving MGCD0103 at higher doses [58].

A phase II trial evaluated mocetinostat in relapsed and or refractory classical HL at doses of 85 mg and 110 mg thrice weekly [59]. Patients were heavily pre-treated and of 21 evaluable patients in the 110-mg cohort, two achieved CR and six PR. The PFS for the patients in CR was 270+ and 420+ days. Five evaluable patients treated at 85 mg, showed a degree of tumor reduction by CT, with one PR and two SD. Serum thymus and activation regulated chemokine (TARC) is highly expressed by Hodgkin Reed-Sternberg (HRS) cells and dendritic cells in the malignant lymph node and is able to provoke a TH2 response [60]. *In-vitro* observations of a reduction in TARC when HL cell lines are treated with vorinostat were recapitulated by the MGCD0103 clinical trial, where responses correlated well with a reduction in TARC levels [59]. It is not clear how the reduction in TARC is achieved and whether its reduction is directly related to anti-tumour efficacy. A phase II study in relapsed or refractory DLBCL or follicular lymphoma (FL) resulted in responses in 17 patients with DLBCL including one CR and three PR, with PFS for responders ranging from 168 to 336+ days. Interestingly, five patients with DLBCL with SD had PFS ranging from 112 to greater than 336 days [61]. Only one of 10 patients with FL achieved a PR. Inhibition of HDAC activity in PBMCs was seen to occur in most patients. 

A phase II clinical trial in CLL began mocetinostat at a dose of 85 mg/d, thrice weekly. All 21 patients had previously received fludarabine, a third of which were fludarabine refractory, with the majority having documented adverse cytogenetics. There were no responses, even in patients receiving permitted dose escalation to 110 mg or the addition of rituximab after two or more cycles. Three patients received 110 mg and four patients received concomitant rituximab, with no improvement in response, despite grade 3-4 toxicities and HDAC inhibition observed in six out of nine patients on day 8 [62]. Whether the clear anti-tumour activity of mocetinostat is unable to overcome the adverse characteristics of patients with 11q or 17p deletions or whether CLL is a disease generally unresponsive to HDACi remains to be seen. 

## 8. Entinostat

Entinostat (MS-275) is a synthetic benzamide derivative HDACi which predominantly inhibits class I HDAC enzymes. A study of entinostat in patients with advanced refractory acute leukemia showed the drug to be well tolerated but did not result in any formal responses, despite 12 patients having brief reductions in peripheral blood blast numbers [11]. Histone and protein hyperacetylation was shown at all dose levels by flow cytometry but marked PK variation at all dose levels made correlation with drug exposure difficult. Indeed the half life was 36 hours, far longer than anticipated, while the AUC did not increase proportionately with drug dose. 

A phase I study combining escalating doses of both 5-azacitidine on days one to ten and entinostat days three and ten, was conducted in patients with advanced myeloid malignancies. Of 27 evaluable patients twelve responded, with two CRs, four PRs, and six hematological improvements. Responses occurred at all dose combinations, in patients with MDS, chronic myelomonocytic leukemia (CMML), and AML. Six clinical responders with baseline cytogenetic abnormalities had a decrease in abnormal metaphases, with four attaining cytogenetic CR [63]. Correlative studies during the first cycle showed no association between patient response and either baseline promoter methylation or its reversal post treatment in bone marrow or the CD34^+^ population. Induction of histone hyperacetylation and the DNA damage–associated variant histone -H2AX was observed in PBMC’s across all dose cohorts. In conclusion, methylation reversal of tumour suppressor genes during cycle 1 of therapy was not predictive of clinical response to combination “epigenetic” therapy [64].

## 9. Expert Opinion

As the more clinically developed HDACi continue to be entered into advanced phase studies, it must be remembered that fundamental questions remain. On the surface this group of drugs appear to have both a class effect in certain diseases, including T-cell lymphomas and HL, a disease with associated profound reactive T-cell tumour infiltrates, and consistent side effect profiles of fatigue, diarrhea and thrombocytopenia. Given the molecularly diverse structure of these compounds, and the knowledge that some compounds are more selective for certain HDACi, does this mean some HDAC enzyme targets are relatively superfluous; are ‘off target’ cytoplasmic targets at least as important for efficacy? Will selective inhibitors be shown to have more disease-targeted activity and reduced toxicities or quite the opposite? Given the PET responses seen in HL, these drugs may have much to tell us about normal T-cell development, recruitment and apoptosis. Mocetinostat is a highly isotype selective HDACi with clear efficacy in HL, with the implication that class I HDACs may be the most important target in cancer, however we have no clinical data to compare it with pan-HDACi such as panobinostat. Direct comparisons of clinical efficacy and biological targets would be fascinating, but are unlikely to occur in the clinical setting in the near future. Ultimately as more HDACi become approved in a variety of indications, we may end up with specific HDACi to be most commonly used for specific diseases. Choice would be presumably be based upon the balance of published data, efficacy, convenience, side effect profile, and capacity to partner other active drugs.

## 10. Future Perspectives

Combination studies are a major focus for HDACi clinical development. Currently, demethylating agents for myeloid malignancies, and bortezomib for myeloma are the most common partner drugs being investigated with Phase III studies underway. Phase II studies in combination with standard chemotherapy are underway for lymphoma (Hodgkin, B-cell, T-cell), firstly in the setting of relapsed disease but will likely move to front-line strategies eventually. Maintenance therapy in minimal residual disease settings is another area of investigation. Of particular interest is whether these well tolerated drugs are able to improve responses and survival times in elderly patients unable to tolerate intensive treatments. 

Extensive pre-clinical data exists for a multitude of other compounds including monoclonal antibodies, tyrosine kinase inhibitors, mTOR inhibitors and BH3 mimetics, so conceivably numerous combination studies will be undertaken for many years to come [75]. 

Despite the barrage of biomarker data demonstrating these drugs are biologically active, such as histone hyperacetylation, further correlative studies remain essential. It may be that specific cytogenetic or molecularly defined sub-groups of patients or tumor HDAC expression can predict for general or specific HDACi response or resistance. Lastly, despite their apparent excellent toxicity profile, these drugs affect the expression of large parts of the genome in both normal and transformed tissues, meaning that long-term monitoring of toxicities is essential, with potential areas of concern lymphocyte, hematopoietic and hormonal function, as well as viral reactivation. 
